# No increased injury incidence in the German Bundesliga after the SARS-CoV-2 virus lockdown

**DOI:** 10.1007/s00402-021-04060-2

**Published:** 2021-07-20

**Authors:** Werner Krutsch, Abed Hadji, Tobias Tröß, Dominik Szymski, Karen aus der Fünten, Barbara Gärtner, Volker Alt, Tim Meyer

**Affiliations:** 1 Department of Trauma Surgery, University Medical Centre Regensburg, Franz-Josef-Strauss-Allee 11, 93053 Regensburg, Germany; 2 Institute of Sports and Preventive Medicine, Saarland University, Saarbrücken, Germany; 3 Department of Medical Microbiology and Hygiene, Saarland University, Homburg, Germany

**Keywords:** Soccer, Pandemic, COVID-19, Injury prevention

## Abstract

**Introduction:**

The coronavirus lockdown in 2020 resulted in a worldwide suspension of professional sports. The first major professional football league to restart after the lockdown was the German Bundesliga. This study investigates whether the injury incidence increased after the restart of the season with only 9 days of regular preparation time and without any friendly matches in comparison to three control periods.

**Materials and methods:**

In a prospective cohort study, injury analysis (at least 1 day of absence from official football matches or training sessions) of the German Bundesliga registry was standardised according to Hägglund et al. (Br J Sports Med 39:340–346, 2005) and Fuller et al. (Clin J Sports Med 16:97–106, 2006) for data collection and to previous publications for the validated use of media sources for injury registration. The study period after the lockdown in May and June of the 2019–2020 season was compared to three control periods: the period directly before the lockdown, the beginning of the 2019–2020 season and the 2018–2019 season final.

**Results:**

The nine match days after the restart of the 2019–2020 season showed an overall injury incidence of 4.9 per 1000 h football. This rate was significantly lower than that of the previous season final (9 last match days, overall injury incidence: 6.9 per 1000 h football; *p* < 0.001) and not increased compared to the rates after the summer break (9 match days; incidence: 5.5/1000 h, *p* > 0.05) or the winter break (8 match days; incidence: 5.6/1000 h, *p* > 0.05).

**Conclusion:**

The period after the unexpected break in the 2019–2020 season due to the coronavirus lockdown and the rapid return to competition showed no increase in the injury rate compared to the pre-lockdown period and a lower injury rate than in the previous season final. The unintentional mid-season rest with its potential for physical recovery and individual fitness training seems to have had a positive effect on injury occurrence.

## Introduction

The worldwide lockdown due to the coronavirus pandemic in March 2020 did not only affect all parts of social life but also suspended sporting activities including professional football. The last pre-lockdown match of the 2019–2020 Bundesliga season took place on 11th March 2020. The season was restarted after 66 days and successfully finished within 42 days [24]. The interruption due to the coronavirus lockdown represented a psychologically and physiologically unique situation for the players. Contact restrictions during the lockdown as well as the implemented hygiene rules interfered with regular training schedules and prevented players from maintaining their normal level of sport-specific fitness.

Many sports medicine experts and practitioners did not believe in the feasibility of a quick restart of professional football [7] after the end of the lockdown period. Yet, the first three men’s professional football leagues as well as the first women’s league in Germany were restarted on the basis of a hygiene concept provided by an expert group of the German Football League (Deutsche Fussball Liga, DFL) and the German Football Association (Deutscher Fussball-Bund, DFB). These hygiene standards—which are available online—mainly consist of a code of behaviour for players and staff, including social distancing, home quarantine, frequent PCR SARS-CoV-2 testing, frequent use of disinfectant and the consequent use of facial masks when not participating in sporting activities [17]. After permission from the German government, regular football training was possible again for the entire team from early May 2020 onwards [24].

One important question after the completion of the 2019–2020 season was whether the injury incidence would be influenced by the players’ long absence from football-specific training and match play and the short-term restart of the league with a tight schedule. Based on the knowledge that short-term changes or abrupt increases in training intensity facilitate severe knee injuries [23], the interruption of the season and the unusual preparation circumstances were suspected to increase severe knee and muscle injuries. Especially the lack of team training for many weeks and the short preparation phase before the first match were considered potential risk factors for severe injuries. Moreover, nine matches had to be played within 42 days, which represented an unfamiliar match load for many players. This constellation might have imposed a higher risk of muscle–tendinous injuries [9].

Therefore, this study investigated the incidence of football-related injuries for the period between the Bundesliga restart on 16th–17th May 2020 and the end of the season 42 days later. Possible changes in injury incidence are best assessed in a valid manner by means of a prospective injury survey, which—for obvious reasons—was impossible in this situation. However, a systematic injury registration was initiated in professional football in Germany 5 years ago. Publications based on this registry have already addressed various topics [5, 6, 13, 22]. The current study investigated whether the period of 9 match days in 42 days between the restart and the completion of the 2019–2020 season in the men’s first professional football league in Germany (Bundesliga) had a higher injury incidence than comparable time periods in previous football seasons.

## Methods

This prospective cohort study investigated football injuries in the men’s first professional league in Germany (Bundesliga) by means of media data obtained from a prospective national injury registry. The analysis only included injury types resulting in at least 1 day of absence from official football matches or training sessions [14]. All injuries sustained in training sessions and official club matches were included.

Injury data provided by medical staff and players as required for longitudinal studies are rarely available. Therefore, injury data for this study were prospectively documented in a standardised manner according to media analysis [14, 15, 22, 23, 27]. This analysis was mainly based upon data obtained from the German kicker^®^ sports magazine, which is published twice a week and represents one of the main media sources about the first Bundesliga in Germany. Each professional football team has one specifically assigned journalist who updates team-specific information. Additionally, injuries were documented and analysed on a daily basis by screening the social media websites (Facebook^®^ and Twitter^®^) of each first league team and their players as well as on online platforms such as www.transfermarkt.de. Every documentation of an injury in the database was followed by a verification process, and each information was confirmed by at least one different source. According to international guidelines, injury diagnoses are best verified by medical staff [14, 15] as recently carried out by this study group [3, 5, 19]. In the current study, we used recently published standards for accurate injury registration by media analysis [18, 22, 27] to confirm the validity of the media-based data (Table 1).

**Table 1:** Quality of media-based injury-specific and football-specific data of football players ( adapted from Krutsch et al. 2019 [22])

Type of information	Media-based information considered valid	Media-based information of questionable validity
Injury pattern	Injured body region (particularly of severe injuries)	Type of injury (particularly of minor injuries)
Anthropometric data	Age, weight and height	–
Football exposure	Match exposure	Training exposure (good estimates are possible)
Injury details	Match and training injury, time of injury and affected leg	Injury mechanism, contact or non-contact injury, foul and concomitant injuries

Injury types were categorised with regard to the affected body region, the type of injury and the time of occurrence during the season according to Fuller et al. [14, 15]. The injury incidence was measured per 1000 football hours (h) of training, matches and overall exposure (training and match injuries combined). Official sports media provide valid information on the match exposure of all football players over the course of a season but not on their training exposure. For this reason, training exposure was estimated on the basis of previous publications by the authors in which the same study population was used in a different study period (seasons 2008–09 and 2009–10) as well as on the basis of the authors’ own experience at professional football levels [3, 20, 27]. Based on these parameters, training exposure was calculated as an average of 7200 h per team per year and 340 h per player per year. The average match exposure amounted to 50 h per season. The study period consisting of the 9 match days between the Bundesliga restart and the end of the 2019–2020 season was compared to three other control periods: the last 9 match days of the 2018–2019 season, the 9 match days after the summer break at the beginning of the 2019–2020 season and the 8 match days after the winter break directly before the lockdown. Besides the overall injury incidence, we also focused on a detailed analysis of typical football injuries, i.e. ligamentous injuries to the knees and ankle joints, musculotendinous injuries to the thighs as well as head injuries.

### Statistical analysis

Because all data on injuries and players were exclusively derived from publicly available media sources, this study did not have to be approved by an ethics committee. Continuous data are expressed as means ± standard deviations (SD) and categorical data as frequency counts (percentages). Proportions between two groups were compared with the Fisher’s exact test and continuous variables with the *t* test. Incidence rates were compared with an exact test based on the Poisson distribution. The significance level was set at *p* < 0.05 for the *α*-error with *p* < 0.01 considered ‘highly significant’. All analyses were carried out with IBM SPSS Statistics, version 24.0 and R (version 3.3.3, The R Foundation for Statistical Computing).

## Results

Overall, 787 injuries occurred during the 2019–2020 Bundesliga season (mean: 43.7 per season per team, overall injury incidence per 1000 h football exposure: 4.64). Mean age of the injured players was 25 years (SD 4.1; Table 2).

**Table 2:** Anthropometric data of the players and their playing position

Anthropometric data	Mean	SD	Min	Max
Age at injury (years)	25.0	4.1	16.0	40.0
Height (cm)	183	6	168	198
Weight (kg)	78.5	6.7	61	98

Playing position	*n* = 497	%
Keeper	36	7.2
Defender	161	32.4
Midfielder	194	39.0
Striker	106	21.3

**Fig. 1:**
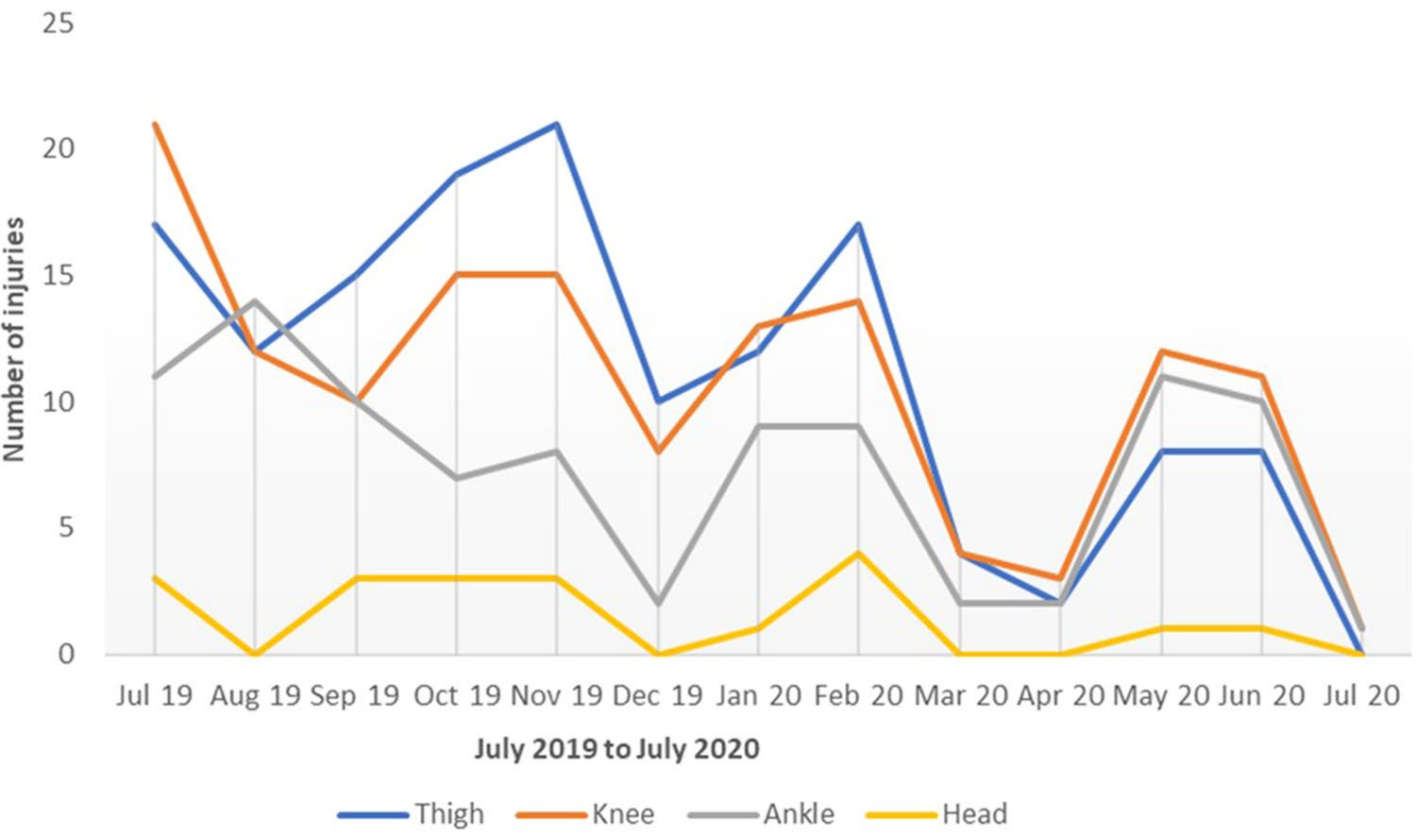
Seasonal distribution of football-related injuries

138 Injuries occurred in the nine remaining match days after the Bundesliga restart (26.5% of all match days), amounting to 17.5% of all injuries in the 2019–2020 season. The body regions most frequently affected by injuries were the knee joints (16.7%) followed by the ankle joints (15.9%), the hip and groin region (13.0%) and the thighs (11.6%; Table 3). The most frequent types of injury were muscle injuries (44.9%) as well as ligament and joint injuries (27.5%). 65.2% of the injuries were sustained during training sessions (Table 3). The injury incidence in the different body regions after the Bundesliga restart showed a decreasing trend over the complete study period (Fig. 1).

**Table 3:** Injury profile in the German Bundesliga after the restart (body region, injury type, injury severity, match or training injury and others)

Injured body region	Category	*n* (138)	% of total (138)
Head and neck	Head or face	3	2.2
Upper limbs	Shoulder or clavicle	1	0.7
Trunk	Sternum, rips or upper back	6	4.4
	Lower back, pelvis or sacrum	6	4.4
Lower limbs	Hip or groin	18	13.0
	Thigh	16	11.6
	Knee	23	16.7
	Lower leg or Achilles tendon	12	8.7
	Ankle	22	15.9
	Foot or toe	11	8.0
Others		20	14.5

Injury type	Category	*n* (138)	% of total (138)
Fractures and bone stress	Fracture	4	2.9
Joint (non-bone) and ligament	Sprain or ligament injury	38	27.5
Muscle and tendon	Muscle rupture, tear, strain or cramps	62	44.9
	Tendon injury, rupture, tendinosis or bursitis	5	3.6
Contusions	Haematoma, contusion or bruise	27	19.6
Central or peripheral nervous system	Contusion (with or without loss of consciousness)	2	1.5

Injury severity	*n* (138)	% of total (138)
Minor (1–3 days)	26	18.8
Slight (4–7 days)	26	18.8
Moderate (8–28 days)	56	40.6
Severe (> 28 days)	30	21.7

Time of injury	*n* (138)	% of total (138)
Match injury	48	34.8
Training injury	90	65.2

**Fig. 2:**
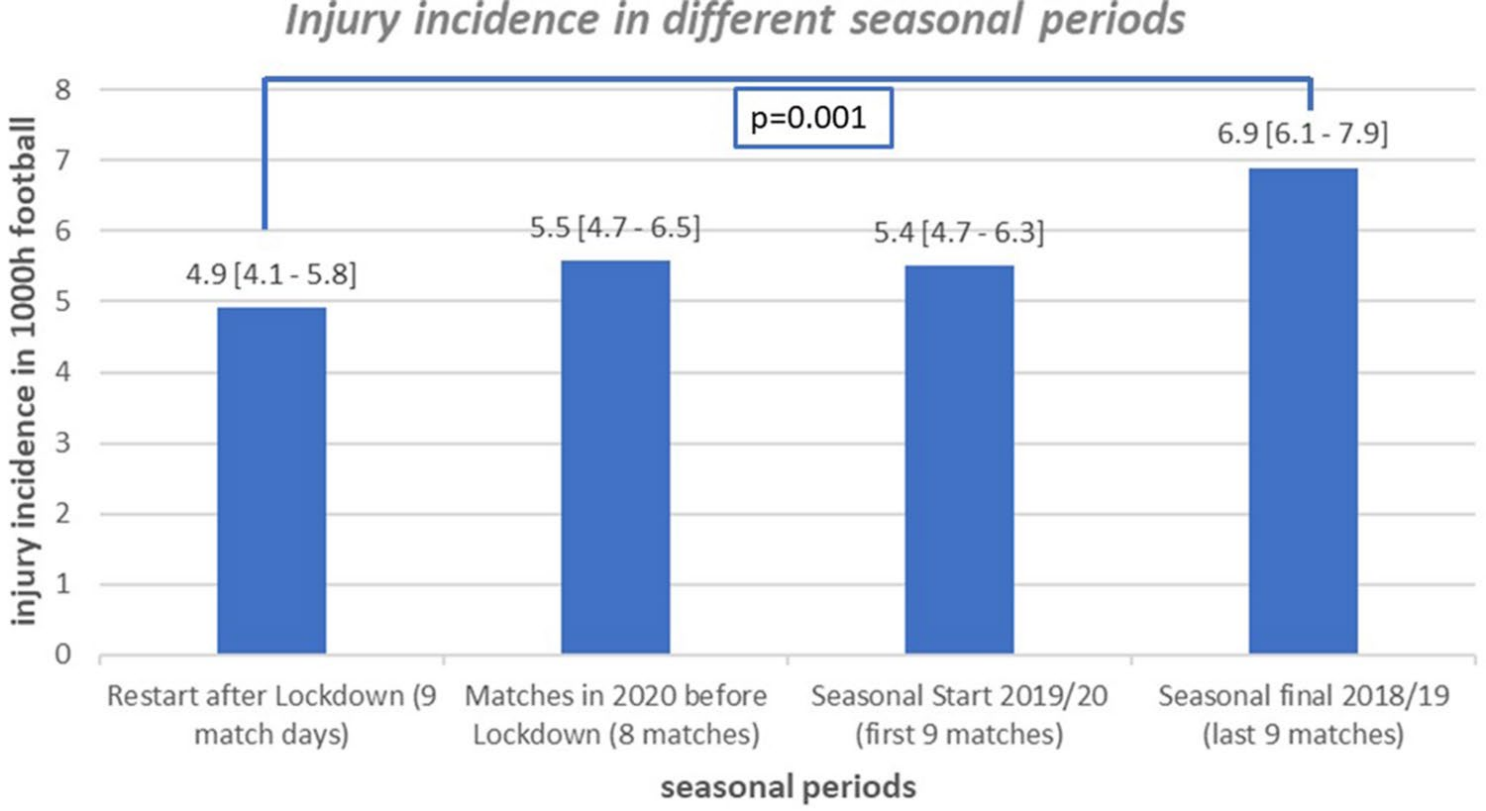
Injury incidence after the restart compared to the rates of three previous seasonal periods

The comparison of the injury incidences between the different periods of the season showed that the overall injury incidence was not higher after the restart than during the preliminary periods of the 2019–2020 season (Fig. 2). The injury incidence in the nine matches after the restart was 4.9 per 1000 h football exposure, which was significantly lower than the rate in the final nine match days of the 2018–2019 season (6.9 per 1000 h; *p* = 0.001). Similarly, the injury incidence in the 42 days after the restart was also not increased compared to the initial weeks after the start of the 2019–2020 season (*p* = 0.308) and after the winter break (*p* = 0.287; Fig. 2).

The incidence rates of muscle and head injuries were lower after the restart of the Bundesliga compared to any other time period. The incidence of knee injuries was similar during all investigated time periods. Only the incidence of ankle injuries was numerically higher after the restart than during either of the other three periods (*p* = 0.89; 0.44; 0.24, Table 4).

**Table 4:** Overall injury incidence and of football-specific injuries according to the different seasonal periods (2018–19 and 2019–20 seasons)

Restart of the 2019–2020 season after the lockdown until the season final (9 match days)			Period between winter break and 2019–2020 lockdown season (8 match days)			Start of the 2019–2020 season (first 9 match days)			Final period of the 2018–2019 season (last 9 match days)		
Body region	Total injury *n* (% of all injuries)	Overall injury incidence per 1000 football hours [95% CI]	Body regio*n*	Total injury *n* (% of all injuries)	Overall injury incidence per 1000 football hours [95% CI]	Body regio*n*	Total injury *n* (% of all injuries)	Overall injury incidence per 1000 football hours [95% CI]	Body region	Total injury *n* (% of all injuries)	Overall injury incidence per 1000 football hours [95% CI]
Thigh	16 (11.59%)	0.57 (0.35–0.93)	Thigh	23 (15.5%)	0.86 (0.57–1.30)	Thigh	52 (19%)	1.46 (1.11–1.92)	Thigh	37 (16.1%)	1.11 (0.81–1.54)
Knee	23 (16.67%)	0.82 (0.54–1.23)	Knee	31 (21%)	1.16 (0.82–1.65)	Knee	27 (13.9%)	0.76 (0.52–1.11)	Knee	27 (11.7%)	0.81 (0.56–1.19)
Ankle	22 (15.9%)	0.78 (0.51–1.18)	Ankle	20 (13.5%)	0.75 (0.48–1.16)	Ankle	22 (9.2%)	0.62 (0.41–0.94)	Ankle	18 (7.8%)	0.54 (0.34–0.86)
Head	3 (2.17%)	0.10 (0.03–0.33)	Head	5 (3.4%)	0.18 (0.07–0.45)	Head	7 (3.1%)	0.19 (0.09–0.41)	Head	6 (3%)	0.18 (0.08–0.40)

## Discussion

For the first time, this study provides data on the impact of the coronavirus pandemic and its associated interruption of training and competition on the injury incidence in professional football. This study compared prospectively collected injury data on the first men’s professional football league in Germany (Bundesliga) obtained from media analysis before and after the lockdown. The nine remaining matches of the 2019–2020 season final played within 6 weeks in May and June 2020 showed significantly fewer injuries than the nine matches of the 2018–2019 season final. The unexpected break in the 2019–2020 season and the rapid return to competition of the first professional sports league worldwide [24], which was controversially discussed before and after the restart, had no negative effect on injury occurrence.

With regard to injury prevention, the time between the restart of team training and matches after the ease of the very strict lockdown restrictions was probably a highly relevant period. None of the 18 Bundesliga teams was allowed to participate in friendly matches with other teams—usually an integral part of pre-seasonal preparation—before the restart of the season. Therefore, the teams had less opportunity of preparing themselves for highly competitive, football-specific, on-field movements or tactics. In terms of injury prevention, practising football-specific movements in competition-like circumstances is deemed crucial [1, 21]. A further aspect to consider is the content of training sessions, and the very short time span between resuming full team training and the start of the nine remaining matches presented a challenge in this regard. According to previous research, short-term changes such as a sudden move to another club, replacement of the coach or relegation to a higher league [23] influence training contents; such influences or just an abrupt start of the season may increase the risk of sustaining some types of football injuries. However, this study does not provide indications for any significant increase in injury incidences after the lockdown and the rapid return to competition.

The unexpected reduction in the injury incidence gives rise to controversial discussions and speculations about the underlying reasons. The players did not completely stop training during the lockdown. Training continued on an individual basis and consisted of endurance, strength and conditioning elements. For some players, the lockdown period may have represented a chance to recover from a long-lasting overuse injury or to cure a minor injury, which may have subsequently led to a major injury. Also, preventive training may have been conducted more frequently and on a more individualised basis than in regular seasons. The decreased injury rate after the Bundesliga restart may have been also based on improved healing processes during the lockdown. This unique situation possibly gave injured tissue adequate time to heal and players the time for structured rehabilitation before their return-to-play [10, 25, 26], which is usually not possible amidst a professional football season. Therefore, previously injured players were able to start playing football with less overuse complaints. Such minor complaints are a risk factor for sustaining more severe injuries to the knees or the ankles [19], and the absence of such injuries led to high match availability after the restart and ultimately maybe to fitter players.

This prospective study showed that the injury incidence after the restart after a much longer mid-season absence from playing football than usual was not higher than in the control periods, i.e. the first few weeks of the 2019–2020 season or the weeks directly before the lockdown. This finding also holds true with regard to muscle injuries that tend to occur more often in case of insufficient fitness or during a tight match schedule [8, 9] such as after the restart with 9 matches in 6 weeks. The somewhat unprepared rapid restart of match play, which depended upon political regulations, made it impossible for the clubs to follow traditional seasonal preparation concepts. Because such short-term increases in psychological and physical stress are well-known factors for sustaining injuries, especially knee injuries [2, 23], this aspect was a major concern before and after the restart. Additionally, the control period of the final weeks of the previous 2018–2019 season showed a higher injury incidence than the 2019–2020 season final after the lockdown. Whether the lower injury incidence in head injuries after the restart was influenced by a lower rate of physical contacts due to a lower frequency of contact duels is speculative and cannot be answered finally. This question should be answered in further investigations based on the video analysis of players’ behaviour and the frequency of contacts or heading duels.

The findings of this study may have an important impact on injury primary, secondary and tertiary prevention strategies in professional football [10, 11]. In comparison to recent studies on injury epidemiology in national professional football in Germany [3, 6, 13, 22], this study shows a comparable injury prevalence with more than 80% of the players and with comparably high injury incidence, especially on muscle and tendon injuries and on joint injuries of the lower extremities [4, 16, 20]. Despite increasing evidence of the efficacy of injury prevention measures and an increasing number of prevention strategies, longitudinal injury surveillance does not show any significant decrease in injury incidences [12]. One reason may be the slow transfer from research into practical routine, which is traditionally difficult to achieve in professional football [21] because time pressure and expectations on short-term success are more important than fundamental injury prevention requiring long-term commitment. Another aspect may be the lack of time for structured injury prevention measures and regeneration during regular seasons because of the tight match schedule or the pressure on teams, coaches and players. However, this study on the restart period after the corona lockdown may indicate that injury incidence can be reduced when football teams take the time for individual preparation strategies as part of primary injury prevention. Other debatable reasons for the lower injury incidence after the restart may be the increase in the number of substitutions from 3 to 5 per match implemented by the FIFA and a generally higher focus of football players on their professional activities because of the restrictions on social life during the pandemic.

### Limitations

One potential limitation of this investigation is the reporting of injuries at the restart of the Bundesliga. The period after the lockdown differed to other seasonal periods: on the one hand, sports journalists were not allowed to follow their routine of being present at the clubs’ training grounds on a daily basis. This absence may be a reason for a potential underreporting of injuries. On the other hand, professional football was the first professional sports to restart with political legitimation after the lockdown. Other professional sports were not allowed to restart, neither was the entertainment industry. This political legitimation increased the attention on Bundesliga football and subsequently on football injuries in the public and sports media and their daily reports about football clubs alike. Media-based injury registry may, therefore, be affected by the overreporting of injuries.

Media-based studies are associated with general limitations that may lead to imprecise injury statistics and debatable conclusions. However, injury reports provided by football clubs during the lockdown may also be invalid due to possible underreporting, but this lack is unlikely to have any impact because of the probably low number of injuries occurred during the lockdown. The current study population was evaluated by means of advanced national, regional and local media analyses with strict exclusion criteria. This media-based analysis was prospectively conducted over 5 years [22, 27], which improved injury recording because the most detailed information can be obtained immediately after the occurrence of an injury. Therefore, the strengths of our approach are the verification of the injury diagnosis by different public sources, its precisely defined methodology and our differentiation between valid and weak information (Table 1). These methods provide media-based data with high validity and represent a huge difference to other uncritically sampled and assessed media data about injuries also available in the scientific literature.

Because of the unexpected onset of the coronavirus pandemic, no other prospective study protocol could be implemented to compare injury occurrence before and after the lockdown. As the injury registration of this study was prospectively conducted for five seasons with constant methodology, the resulting injury incidence after the restart should be comparable to other periods of the present and previous seasons alike. The direct comparison of the 2019–2020 season final after the restart to the preceding 2018–2019 season final seems reasonable. Because of the lockdown break during the 2019–20 season, these two-time intervals differ significantly in physical preparation and also in a higher match frequency in a shorter period of time. Thus, the differences in the preparation before the last nine matches of the 2019–2020 season and the matches of the 2018–2019 season could be a reason for the significantly lower injury incidence. However, because the study period after the restart followed a longer break, we additionally used the periods after the summer and winter break as controls. Further investigations should follow based on our data. Especially the aspects fitness and training programmes during and after the lockdown may be important fields of research in the future.

## Conclusion

The period after the unexpected break in the 2019–2020 Bundesliga season due to the coronavirus lockdown and the rapid return to competition showed no increase in the injury rate compared to the post-lockdown period and a lower injury rate than in the previous season final. The unintentional mid-season rest with the potential for physical recovery and individual fitness training seems to have had a positive effect on injury occurrence.
